# SLC13A5 Deficiency Disorder: From Genetics to Gene Therapy

**DOI:** 10.3390/genes13091655

**Published:** 2022-09-15

**Authors:** Kimberly Goodspeed, Judy S. Liu, Kimberly L. Nye, Suyash Prasad, Chanchal Sadhu, Fatemeh Tavakkoli, Deborah A. Bilder, Berge A. Minassian, Rachel M. Bailey

**Affiliations:** 1 Division of Child Neurology, Department of Pediatrics, University of Texas Southwestern, Dallas, TX 75390, USA; 2 Warren Alpert School of Medicine, Brown University, Providence, RI 02903, USA; 3 TESS Research Foundation, Menlo Park, CA 94026, USA; 4 Department of Research & Development, Taysha Gene Therapies, Dallas, TX 75247, USA; 5 Division of Child & Adolescent Psychiatry, Department of Psychiatry, University of Utah, Salt Lake City, UT 84108, USA; 6 Center for Alzheimer’s and Neurodegenerative Diseases, University of Texas Southwestern, Dallas, TX 75390, USA

**Keywords:** epileptic encephalopathy, SLC13A5, sodium/citrate cotransporter, gene therapy, AAV9

## Abstract

Epileptic encephalopathies may arise from single gene variants. In recent years, next-generation sequencing technologies have enabled an explosion of gene identification in monogenic epilepsies. One such example is the epileptic encephalopathy SLC13A5 deficiency disorder, which is caused by loss of function pathogenic variants to the gene SLC13A5 that results in deficiency of the sodium/citrate cotransporter. Patients typically experience seizure onset within the first week of life and have developmental delay and intellectual disability. Current antiseizure medications may reduce seizure frequency, yet more targeted treatments are needed to address the epileptic and non-epileptic features of SLC13A5 deficiency disorder. Gene therapy may offer hope to these patients and better clinical outcomes than current available treatments. Here, we discuss SLC13A5 genetics, natural history, available treatments, potential outcomes and assessments, and considerations for translational medical research for an AAV9-based gene replacement therapy.

## 1. Introduction

Developmental epileptic encephalopathies (DEE) are a group of disorders in which developmental disability persists despite attaining adequate seizure-control. With growing access to clinical genetic testing, causative monogenic etiology for many DEEs have been identified. One such condition is SLC13A5 deficiency disorder (SDD). In this disorder, neonatal seizures are often the initial presenting feature, but developmental language and motor impairments develop and persist into adulthood despite improved seizure-control with approved anti-seizure medications [1,2,3,4,5]. The degree of neurodevelopmental disability and seizure-burden can vary, but most adults are unable to live a fully independent life [5].

The gene *SLC13A5* coding for the plasma membrane sodium-coupled citrate transporter (NaCT) is highly expressed in brain, teeth, liver, and testes [5]. Within the brain, NaCT is found in neurons and is likely to be involved in the regulation of astrocyte-released citrate as a means of modulating N-methyl-D-aspartate receptor (NMDAR) activity [6,7]. Pathogenic loss of function variants in *SLC13A5* impair NaCT function, leading to decreased citrate inside of neurons and result in seizures within the first week of life [4]. The resulting bi-allelic epileptic encephalopathy is known as SLC13A5 deficiency disorder (SDD) and may also be referred to as developmental epileptic encephalopathy-25, early infantile epileptic encephalopathy-25, SLC13A5 epilepsy, SLC13A5 Citrate Transporter Disorder, Citrate Transport Disorder, SLC13A5 Deficiency, or Kohlschutter-Tonz Syndrome (non-ROGDI) [4,5,8,9].

For monogenic epilepsies, such as SDD, advances in the field of gene therapy present the opportunity to address the underlying pathogenesis of disease. Here, we discuss SLC13A5 genetics, natural history, available treatments, and considerations for translational research, for an AAV-based gene replacement therapy for SDD.

## 2. Genetics and Molecular Basis

The cause of SDD was first established in 2014 by whole exome sequencing of three individuals with early onset epileptic encephalopathy, whose first seizures occurred within the first week of life [4]. That investigation revealed three variants in *SLC13A5* across three families that affected either the first or second sodium binding site of NaCT, suggesting that SDD in these patients was the result of impaired citrate transport into neuronal cells due to sodium binding deficiency.

To date, more than 40 known *SLC13A5* variants that lead to epilepsy (including nonsense and missense variants, frame shift variants from single nucleotide polymorphisms, exon deletions, and whole gene deletions) are bi-allelic, with c.655G>A (p.G219R) and c.680C>T (p.T227M) being the most common [10,11]. Individuals with SDD may be homozygous for the loss of function variants of *SLC13A5* or have compound heterozygous variants. Individuals with the same variants vary in seizure frequency and developmental disability, indicating heterogeneity of the disease and possible involvement of modifier genes [1,5].

The exact mechanism by which impaired NaCT function results in SDD is unknown. NaCT preferentially transports citrate and can mediate uptake of other carboxylates, such as succinate, albeit at a much lower affinity [12]. Citrate is the main tricarboxylic acid pathway intermediate and is thus involved in energy production, so intracellular deficiency of citrate could alter energy production and result in SDD-associated brain dysfunction. Citrate is also involved in glutamate/γ-aminobutyric acid (GABA) synthesis and may alter the homeostasis of these neurotransmitters [13]. Additionally, citrate has a role in fatty acid synthesis [14]. Therefore, non-physiological levels of citrate may result in delayed myelination seen in some patients, which could impact neuronal function and brain structure [15]. Another known function of citrate is zinc (Zn^2+^) chelation. The Zn^2+^ ion directly inhibits NMDARs; therefore, impaired citrate uptake may lead to excess extracellular Zn^2+^ and greater NMDAR-mediated postsynaptic activity in neurons [7,15,16].

The mouse NaCT has a Michaelis-Menten constant (K_M_) of ~40 µM for citrate, whereas the human NaCT has a substantially higher constant of ~650 µM for citrate [17,18]. At physiological concentrations of citrate, 150–200 µM for both mouse and human, the mouse NaCT functions in a saturated manner, whereas the human NaCT does not. This may suggest physiological differences in citrate metabolism between these species and why *Slc13a5* knock out (KO) mice have a milder phenotype than SDD patients [19,20,21].

## 3. Natural History

The phenotypic spectrum of SDD is reviewed in Table 1. Seizure onset in patients with SDD often occurs within the first week of life [4]. One study found 22 of 23 SDD patients developed seizures within the first week of life with the 23rd developing seizures within the first year of life [5]. Seizure semiology varied between patients with generalized tonic-clonic being the most common type (74% of cases) [5]. Patients may go on to experience status epilepticus within the first month of life but often gradually reach seizure freedom with the aid of optimally-dosed antiseizure medication (ASM) by 18 years of age [22]. While patients may die from SDD (7 of 45 patients from 22 families reviewed previously), SDD patients may live into their 50 s or longer [22,23].

In addition to seizures, affected individuals have global developmental delay, intellectual disability, psychomotor impairments, and lack of speech [1,4]. Dental issues are also common, namely teeth hypoplasia and hypodontia (likely given citrate’s important role in stabilizing bone nanocrystals), yellow to orange discoloration of teeth, abnormal crown formation, wide interdental spaces, and gingival hyperplasia [2,3,22,24,25]. These tooth abnormalities affect both primary and secondary teeth and may limit patients to a soft diet [26]. A study of 15 patients found no differences from peers in height, weight, or head circumference in the first 3 years of life [10]. However, a study of 23 patients (ranging in age from 3–353 months) found patients’ mean weight was at the 32.7 percentile and the mean height was at the 37.9 percentile, suggesting some growth attenuation in affected individuals that may occur at later ages [5].

Electroencephalography (EEG) and brain magnetic resonance imaging (MRI) can reveal non-specific abnormalities in patients with SDD. Interictal EEG can be normal, but other features such as intermittent background slowing or rhythmic delta-theta slowing have been reported. Epileptiform discharges including generalized spike-wave, polyspike-wave, multifocal sharps or spike-waves, and focal spike-wave are common [1,2,3,4,5]. Many individuals with SDD have normal brain MRIs [3,27], but some patients may present with punctate white matter lesions on magnetic resonance imaging of the brain at 6 months of age and subsequent gliosis and delayed myelination [15]. Focal cortical dysplasia has also been documented in a patient with SDD [27].

## 4. Treatments

Seizure control is the main focus of current treatments. GABAergic ASM, such as stiripentol, which increases GABA neurotransmission, and acetazolamide, a carbonic anhydrase inhibitor and atypical ASM, were found to decrease seizures in some SDD patients [3,28]. Through empirical approaches, there seems to be some success in seizure management using a combination of acetazolamide and valproic acid [29]. This suggests that while seizures are severe in infants, seizure control with one or more ASMs is a realistic goal for many as children age [4,22]. However, while ASMs reduce seizure frequency in SDD patients, they do not address associated motor, speech, and cognitive impairments, highlighting critical needs for further innovation [3]. Interestingly, the ketogenic diet has mixed results in SDD, as it improves symptoms in some, but made symptoms worse in others [2,3]. The only treatments available for the developmental disability are therapies such as speech therapy, occupational therapy, physical therapy, feeding therapy, behavioral therapy, and durable medical equipment to aide with functional mobility. Many individuals may also use an augmentative and alternative communication device to communicate.

## 5. Gene Therapy as a Potential Treatment

Gene therapy is a precision medicine where functional genetic material is delivered to cells that has the potential to address the underlying cause of a disorder rather than managing symptoms. Adeno-associated virus (AAV) gene replacement therapy has achieved success in CNS disorders, such as spinal muscular atrophy (SMA) [30] with the approval of onasemnogene abeparvovec-xioi (Zolgensma; Novartis Gene Therapies, Bannockburn, IL, USA) in 2019. Additionally, many AAV gene therapy clinical trials are underway for other loss of function monogenic disorders and such approaches could be applied to address SDD. While AAV vectors are small, with a packaging size < 5 kilobases, the coding sequence of the *SLC13A5* gene is within this limit, making gene therapy an amenable approach [31].

### 5.1. Preclinical Considerations

Preclinical development of an AAV-based gene therapy can be complex from the initial design of the construct to utilizing relevant disease animal models to optimize the viral vector dose, route of administration, and age or stage of disease progression at which treatment is effective [32]. Among the different serotypes of AAVs, AAV9 can cross the blood–brain barrier relatively more efficiently, transduce neurons and glial cells, and has previously been used as a vector for gene therapy for SMA [30,31] and other rare neurological disorders, such as GAN (NCT02362438) [33]. As such, an AAV9 gene replacement therapy is a feasible treatment approach for SDD and is currently under development (Bailey, unpublished work).

Animal models of disease may be particularly challenging for recently identified genetic epilepsies, as these models may not yet be developed or fully characterized. In the case of SDD, there is an *Slc13a5* KO mouse model that is lacking NaCT and shows increased neuronal excitability and propensity for epileptic seizures, significant alterations of citrate and other metabolite levels in cerebrospinal fluid (CSF) and brain tissue, and impaired bone and tooth development [20,34,35,36]. As such, *Slc13a5* KO mice can be used to confirm NaCT protein expression and localization to the plasma membrane and vector distribution within the brain and throughout the body. Additionally, changes in citrate and other metabolite levels, brain activity, and seizure susceptibility can be assessed to determine if a treatment is beneficial and inform on critical parameters such as dosing, route of administration and age of treatment.

While *Slc13a5* KO mice model salient aspects of SDD that can be utilized to develop an AAV9 SLC13A5 gene replacement therapy, they do not show signs of overt behavioral or cognitive impairment seen in patients with SDD [3]. These differences between mice and humans may be indicative of underlying biochemical (e.g., different K_M_) or physiological differences in NaCT function between species [37,38]. This difference may limit the applicability of the *Slc13a5* KO mouse model and there is need for further development of SDD animal models that recapitulate additional clinical symptoms.

### 5.2. Safety Considerations

In addition to preclinical proof-of-concept studies in biologically relevant animal models, pharmacological and toxicology studies are warranted to find the optimal viral vector dose to be safely delivered to humans [39]. *Slc13a5* gene expression in the rat cerebral cortex was shown to be low at birth and increased in the days following birth, which raises a concern that high NaCT levels during the prenatal or neonatal period may impact proper brain development [6]. Indeed, in transgenic mice, overexpression of SLC13A5 in a subpopulation of neurons (CAMKIIa+) beginning during embryonic development was found to result in autistic-like and jumping behaviors, altered white matter integrity and synaptic plasticity, and aberrant synaptic structure and function [40]. While an AAV9-based gene therapy to treat SDD would be administered postnatally and target multiple neuronal types and glia, it could still cause SLC13A5 overexpression. As such, the optimal age at which animal models receive gene replacement therapy should be explored to maximize efficacy while minimizing adverse effects.

Toxicity studies conducted in wild-type mice, rats, and/or larger animals, such as nonhuman primates, provide further reassurance of safety prior to initiation of a clinical study. Here, differences in NaCT structure and function between species may also impact interpretation and translation of findings. Rodent NaCT has a much greater affinity for citrate than the human and non-human primate counterparts. Additionally, lithium inhibits NaCT in rodents, but stimulates NaCT in primates, suggesting species-specific biochemical differences in the transporter [37]. The development of gene replacement therapy for SDD should therefore place emphasis on non-human primate toxicological investigations and include neurological assessments before advancing to clinical trials.

### 5.3. Clinical Research and Outcome Assessments

Only a single compassionate use trial (NCT02500082) has investigated a possible treatment specific to SDD. In this trial, investigators followed the clinical course of subjects receiving triheptanoin (UX007), a substrate reduction therapy. Data from this trial has not yet been published, and the trial is no longer active.

Regulators are increasingly prioritizing patient-focused drug development and have developed guidance on incorporating the patient perspective into the drug development process [32]. In preparation for new human studies, researchers are advised to work with patients, caregivers, patient organizations, and advocacy groups, to inform the clinical trial design. Potential outcome measures include assessment of domains such as seizures, motor, cognitive and adaptive functioning, speech delays, caregiver/family burden, biomarkers, and sleep. Assessment of both patient-centered outcome measures and surrogate biomarkers will likely be needed in future clinical trials.

Because epilepsy is a prevalent feature of SDD, a seizure-related outcome measure may be incorporated for efficacy assessment of potential therapeutics. Seizures may be clinically assessed by changes in seizure activity (duration, frequency, severity), effect of ASM, and electroencephalogram epileptiform activity. The Pediatric Quality of Life epilepsy module can also be used to measure health-related quality of life in children with epilepsy. This module, with both child- and caregiver-report versions available, contains 29 items with five subscales: impact, cognitive, sleep, executive functioning, and mood/behavior [41]. However, because the seizure-burden appears to vary by age of the individual (highest burden during infancy with gradual reduction thereafter), it may be challenging to demonstrate a treatment benefit in older individuals with SDD if outcome measures are limited to seizure burden.

A significant unmet clinical need in SDD is the neurodevelopmental aspects of the phenotype. SDD is known to cause significant delay in cognitive and motor development, and a gene therapy that targets the CNS broadly may have the potential to modify the disease progression in these domains. Disease-specific outcome measures, while ideal, are rarely available for rare diseases. This is especially true for SDD, as this disorder was recently identified [4]. However, there are many assessment tools available to measure global developmental and adaptive abilities among individuals experiencing a wide range of impairment. These developmental assessments include the Vineland Adaptive Behavior Scales, third edition (Vineland-3), the Bayley Scales of Infant and Toddler Development (BSID), the Mullen Scales of Early Learning (MSEL), the Differential Ability Scales-II (DAS-II), or the Peabody Developmental Motor Scales, second edition (PDMS-2) [42,43]. Though none of these measures have been validated in the SDD population, some have been used in other genetic DEEs. Adapted versions of the MSEL have been validated in children with Rett Syndrome up to 10 years of age [44].

### 5.4. Biomarker and Other Assessments 

Numerous biomarkers with roles in inflammation, hypoxia, oxidative stress, and proteins or amino acids with other roles are being investigated for epilepsies. No single biomarker will likely serve as a meaningful indicator of disease severity or treatment responses across all epilepsies given differences in underlying pathologic mechanisms. Sonnewald et al. demonstrated that large amounts of citrate are released by astrocytes and taken up by neurons within the CNS through NaCT activity [45], suggesting that CSF citrate levels within human biological samples may be expected to be increased in SDD-affected individuals. Indeed, increased citrate levels in CSF have been reported in the mouse *Slc13a5* KO model, as well as affected patients [1,20]. For SDD, citrate levels in biological samples (CSF, plasma, and urine) have the potential to be an effective biomarker, though more studies are needed. Homeostasis of other metabolites of the citric acid cycle and downstream neurotransmitters (e.g., glutamate) are disrupted in CSF and could potentially define a metabolic signature for SDD [1,36].

Cardiac, gastrointestinal, renal, and breathing-related sleep disorders, are also identified in patients with SDD and may impact the quality of life of both the patient and caregiver [10]. Sleep changes can objectively be measured by the proportion of slow-wave activity during nonrapid eye movement sleep. Additionally, sleep delays can also be assessed with observer-reported communication and passive data collection from wearable devices.

## 6. Conclusions

SDD is a monogenic epileptic encephalopathy characterized by neonatal seizures that tends to respond to ASM, while other debilitating symptoms remain unaddressed. As the gene (*SLC13A5*) and its loss of function pathogenic variants for this disease are now identified, there is strong rationale for the development of a gene replacement strategy to treat SDD. Challenges for gene therapy development include limitations in currently available disease models and knowledge gaps related to disease mechanisms and natural progression. Clinical trials will need to be thoughtfully designed to demonstrate a meaningful and significant clinical improvement, while minimizing risk for SDD patients.

## Figures and Tables

**Table 1:** SLC13A5 deficiency disorder phenotype—Summary of the phenotypic spectrum reported in the published literature of cases aged 9-months to 29-years. Developmental disability and epilepsy are the cardinal features of SDD. Language impairment is severe with relative sparing of receptive communication and motor disabilities are prevalent. Epilepsy typically starts in the neonatal period with increased variability of seizure-type, seizure-frequency, and EEG abnormalities throughout life. Brain imaging is largely non-diagnostic.

Attribute	Clinical Presentation
Developmental Delay	Moderate to severe general developmental delay Severe expressive speech delay Relative sparing of receptive language abilities Moderate to severe motor delay Intellectual disability Autistic traits in some
Motor Phenotype	Severe motor incoordination Choreoathetosis Dystonia Dyskinesia
Epilepsy	Nearly all; onset within 1st week of life Status epilepticus common in neonates Variable seizure frequency from daily to seizure-free in childhood GTC most common Other types: subclinical, focal clonic, absence, tonic, spasms
EEG Abnormalities	Background slowing Generalized spike-wave Multifocal spike-wave Polyspikes Focal spike-waves Some have normal interictal backgrounds
MRI Abnormalities	Most are normal Periventricular leukomalacia Chiari I Neonatal subdural hematoma Punctate white matter hemorrhage Focal cortical dysplasia Delayed myelination
Other Features	Tooth abnormalities Hypotonia Growth attenuation Feeding problems or dysphagia Gastrointestinal problems Genitourinary problems Musculoskeletal problems Respiratory insufficiency Cardiac anomalies

## Data Availability

Not applicable.
